# Development of a multiclass method to quantify phthalates, pharmaceuticals, and personal care products in river water using ultra‐high performance liquid chromatography coupled with quadrupole hybrid Orbitrap mass spectrometry

**DOI:** 10.1002/ansa.202000015

**Published:** 2020-09-23

**Authors:** Deviprasad Rendedula, Gubbala Naga Venkata Satyanarayana, Ankita Asati, Muralidharan Kaliyaperumal, Mohana Krishna Reddy Mudiam

**Affiliations:** ^1^ Analytical and Structural Chemistry Department CSIR‐Indian Institute of Chemical Technology Tarnaka, Uppal Road Hyderabad 500007 India; ^2^ Academy of Scientific and Innovative Research (AcSIR) Ghaziabad 201002 India; ^3^ Discovery Analytical Sciences Division GVK Biosciences Hyderabad 500007 India; ^4^ Analytical Chemistry Laboratory Regulatory Toxicology Group CSIR‐Indian Institute of Toxicology Research Lucknow 226001 India; ^5^ Department of Chemistry School of Applied Sciences Babu Banarasi Das University Lucknow 226028 India

**Keywords:** multiclass method, organic micropollutants, PPPCPs, solid‐phase extraction, UHPLC‐Q‐Orbitrap‐MS

## Abstract

**Rationale:**

The organic micropollutants such as phthalates, pharmaceuticals, and personal care products (PPPCPs) enter the surface water through various routes. The aim of this study is to develop a sensitive and efficient method to identify and quantify 26 PPPCPs found in river water with acceptable accuracy and precision using a liquid chromatograph hyphenated with quadrupole hybrid Orbitrap mass spectrometry (Q‐Orbitrap‐MS) in a single chromatographic run.

**Method:**

The organic micropollutants were extracted from river water by solid‐phase extraction (SPE) using hydrophilic‐lipophilic balance sorbent and analyzed using an ultra‐high performance liquid chromatograph (UHPLC) equipped with C_18_ stationary phase for chromatographic separation. The targeted mass experiments were conducted in a Q‐Orbitrap‐MS system in positive and negative electrospray ionization mode.

**Results:**

The method was found to be linear in the concentration range of 1‐125 ng/L with coefficient of determination lying in the range of 0.995‐0.999. The method achieved limit of quantification in the range of 0.41‐1.72 ng/L, and method recovery measured at three different concentrations was found to be in the range of 75‐115%. Intra‐ and interday precision expressed as percent relative standard deviation was found to be <15%. Matrix effect was found to be in the range of 83.5‐109.79%. The matrix match calibration was used for quantification of PPPCPs in river water sample. The method performance was evaluated by analyzing real samples collected from Ganga River, and the concentrations of 21 analytes were found to be in the range of 0.76‐9.49 ng/L for pharmaceuticals, 1.49–8.67 ng/L for phthalates, and 0.9‐7.58 ng/L for personal care products.

**Conclusions:**

The present method was found to be precise, sensitive, and rapid to determine 26 PPPCPs including phthalates in river water samples using SPE‐UHPLC‐Q‐Orbitrap‐MS.

**Abbreviation**
AGC: automatic gain control
GPS: global positioning system
HCD: higher energy collisional dissociation
HESI: heated electrospray ionization source
HLB: hydrophilic‐lipophilic balance
HRMS: high resolution mass spectrometry
LLE: liquid‐liquid extraction
ME: matrix effect
PPCPs: pharmaceuticals, and personal care products
PPPCPs: phthalates, pharmaceuticals, and personal care products
PRM: parallel reaction monitoring
Q‐Orbitrap‐MS: quadrupole hybrid Orbitrap mass spectrometry
SPE: solid‐phase extraction
UHPLC: ultra high‐performance liquid chromatography

## 1 INTRODUCTION

River water is an essential source of drinking water for both rural and urban communities. However, pollution of rivers is a global issue that has to be dealt seriously by identifying the pollutants and ways to detect them. A large number of anthropogenic pollutants (pharmaceuticals and personal care products, PPCPs) are being discharged from industrial, commercial, domestic, and agricultural sources leading to surface water contamination, which in turn not only threatens aquatic life but also affects human health.^1^, ^2^, ^3^, ^4^, ^5^ Attempts have been made by several researchers to remove water contaminants and the efforts to decontaminate water are still being explored.^6^, ^7^ Although the majority of micropollutants are present at low levels in the surface water, they can increase health hazards to both flora and fauna in the aquatic atmosphere and also indirectly to the terrestrial habitat. While their long‐standing effects on living beings are mostly unknown, their harmful impact cannot be ignored.^1^, ^8^ The monitoring of river water for organic or inorganic micropollutants has drawn the attention of researchers to understand their contamination levels and design mitigation strategies.^7^, ^9^, ^10^ The PPCPs, known as organic micropollutants, are being extensively used in daily life and are a cause of concern because they are being continuously discharged into the aquatic environment.^8^, ^9^, ^11^, ^12^ PPCPs have been broadly classified into different groups based on their structural and physicochemical properties and one needs to understand their impact on human health.^13^, ^14^, ^15^ Several methods are available for analysis of PPCPs in various environmental samples, but no method has been reported for the simultaneous high‐throughput quantitative analysis of 26 targeted pharmaceuticals and personal care products including phthalates (PPPCPs) by solid‐phase extraction‐ultra high performance liquid chromatography‐quadrupole hybrid orbitrap mass spectrometry (SPE‐UHPLC‐Q‐Orbitrap‐MS) in water samples in a single chromatographic run. The analysis of these different classes of chemicals requires an efficient analytical method that is able to identify and quantify them at low concentration with acceptable accuracy and precision.

Recently, rapid advancements in analytical techniques have reduced the detection limits to nanogram levels with high precision. Several methods are available based on gas chromatography‐mass spectrometry (GC‐MS) for the analysis of PPCPs in river sediments,^16^ sewage sludges,^17^ groundwaters,^18^ surface water,^19^ and aquatic plants.^20^ But they have limitations like low sensitivity and require additional derivatization step to analyze analytes with polar functional groups.^21^ Another hyphenated analytical technique like liquid chromatography‐mass spectrometry (LC‐MS) uses different mass analyzers like triple quadrupole used in analysis of sediment,^22^ time of flight used for water,^23^ waste water,^24^, ^25^ and linear ion‐trap used for water,^26^ surface water,^27^ and environmental waters^28^ as well as quadrupole‐Orbitrap used for surface water,^29^, ^30^ waste water,^31^, ^32^ soil, and plants^33^ for the PPCPs analysis. Although LC‐triple quadrupole‐MS has played an important role in the identification and quantification of targeted analytes,^9^ it has limitations for the identification of PPCPs in the untargeted analysis due to its low mass resolution (unit mass). Due to, higher mass accuracy and precision, LC coupled with high‐resolution MS can be used for the analysis of both targeted and untargeted analysis of PPCPs.^31^

The monitoring of these organic micropollutants in environmental samples like water, soil, and sediment requires an effective extraction method to remove the matrix interferences and enrich the low concentration of analytes from a large volume of water sample.^34^ Owing to the diverse chemical nature and polarity of the selected analytes, liquid‐liquid extraction (LLE) and solid‐phase extraction (SPE) are suitable for extraction and clean‐up of multiclass analytes. The disadvantages of LLE are: needs a large volume of sample as well as solvent, has low accuracy, and is a time‐consuming process.^35^ The advantages of SPE method as compared to LLE are (a) availability of a wide range of sorbents for extraction of various analytes, (b) eco‐friendly, efficient, cost‐effective, and high recovery rate, and (c) needs less solvent for extraction. All these advantages of SPE method make it a better choice for extraction and clean‐up of PPPCPs from environmental samples.^25^ The main aim of the study is to develop, validate, and evaluate the performance of the SPE‐UHPLC‐Q‐Orbitrap‐MS system for the simultaneous determination of 26 PPPCPs including phthalates in river water samples.

## 2 MATERIALS AND METHODS

### 2.1 Chemicals and reagents

A total of 26 PPPCPs (7 phthalates, 12 pharmaceuticals, and 7 personal care products) were purchased from Sigma‐Aldrich (St. Louis, MO, USA) with >97‐99% purity range. The mass spectrometric grade water, methanol, and acetonitrile were purchased from Optima Fisher Scientific USA (New Jersey, USA), and mass spectrometric grade formic acid was purchased from Merck (Darmstadt, Germany). Oasis hydrophilic‐lipophilic balance (HLB) SPE cartridges (3 g, 6 mL) were purchased from Waters (Milford, MA, USA) and filter paper (0.22 μ) was purchased from Millipore.

### 2.2 Standard preparation

The stock solutions of individual standards were prepared at a concentration of 1.0 mg/mL with methanol as a diluent. A total of 1 μg/mL of mixed standard solution was prepared by taking stock solutions of each analyte in a mixture of water:methanol (50:50, v/v) and all standards were stored in a glass volumetric flask at ‐20°C until use.

### 2.3 Sample collection

**FIGURE 1:**
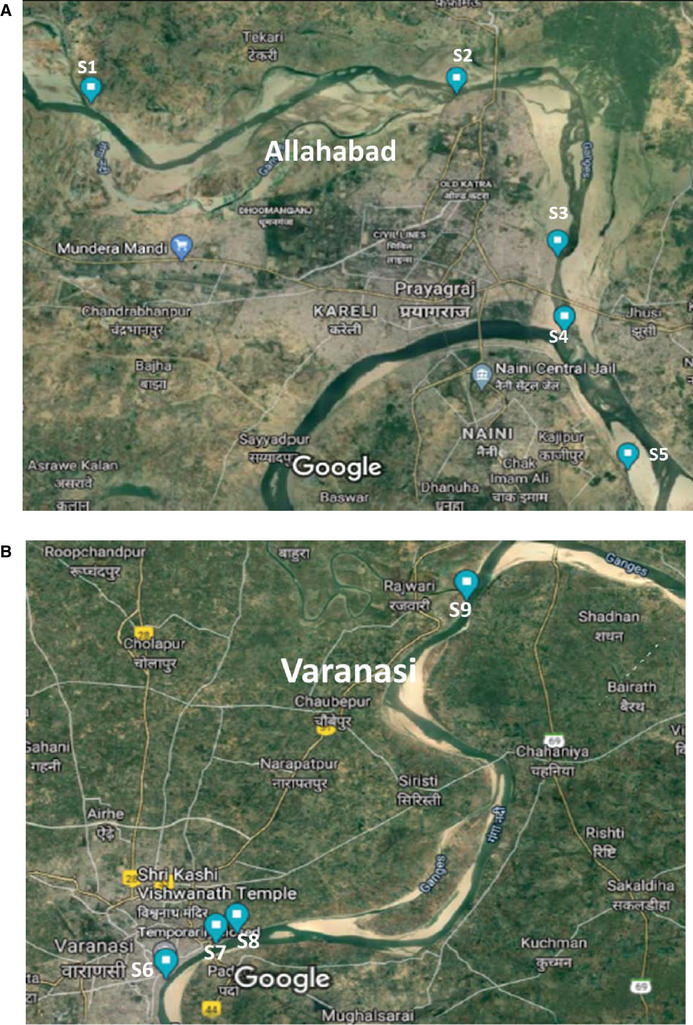
**A**, GPS map of Ganga river point at Allahabad (S1‐S5). **B**, GPS map of Ganga river point at Varanasi (S6‐S9)

The water samples were collected from the River Ganga at nine different points of Allahabad and Varanasi, Uttar Pradesh, India using global positioning system (GPS) coordinates as shown in Table 1. Figure 1A display GPS map of Ganga River points (S1‐S5) in Allahabad, and Figure 1B shows Ganga River points (S6‐S9) in Varanasi. The collected samples were brought to the laboratory in amber color glass bottles under ice‐cold conditions, and filtered through Millipore filter paper. The pH of the samples was adjusted to 3.0 with formic acid to reduce microbial growth and then the samples were stored at ‐20°C until analysis.

**TABLE 1:** GPS coordinates of different sampling points in India

Sample number	Sampling points	Latitude	Longitude
S1	Kuresar ghat, Allahabad	25.498340	81.735181
S2	Rasoolabad ghat, Allahabad	25.501465	81.853328
S3	Daraganj ghat, Allahabad	25.449306	81.886168
S4	Chitkana ghat, Allahabad	25.381157	81.908793
S5	Sangam, Allahabad	25.425045	81.888219
S6	Sheetla ghat, Varanasi	25.307168	83.011086
S7	Raj ghat, Varanasi	25.325142	83.037073
S8	Varuna River, Varanasi	25.330105	83.047934
S9	Markendeya Mahadev ghat, Varanasi	25.500890	83.167124

### 2.4 Sample preparation

The SPE of PPPCPs from the river water samples was performed using Waters Oasis HLB cartridge as the sorbent for maximum extraction efficiency because it can extract a wide range of analytes at different pH levels.^19^, ^25^ The SPE conditions were optimized using river water samples spiked with analytes at a concentration of 50 ng/L. Before loading the samples in SPE cartridges, the cartridges were preconditioned with 5 mL of methanol and 5 mL of ultra‐pure Milli‐Q water. The water samples (3.0 L) were loaded on to the cartridges at the flow rate of 5 mL/min, and then cartridges were air‐dried under vacuum. Ten milliliter mixture of dichloromethane: methanol (1:1, v/v) was eluted with sorbent for the obtaining maximum recovery of the PPPCPs. The extracted organic phase was dried in a nitrogen evaporator (TurboVap RV) and finally, the dried aliquot was reconstituted with 2000 μL of water:methanol (50:50, v/v) for further analysis.

### 2.5 Blank sample

A blank sample was used to detect possible contamination during analysis. To avoid contamination, the following preventive procedure was followed: (a) Plastic materials were not used in sample collection, preservation, preparation, and analysis; (b) Standards and samples were prepared in amber color glassware to prevent possible contamination from air and degradation from temperature and light; (c) All glassware were cleaned with acetone, then dried before analysis at 250°C for 2 h in an oven; (d) Only PTFE septa, filter, and metal tubing were used in UHPLC‐Q‐Orbitrap‐MS; (e) The cross‐contamination was monitored with solvent blank and sample blank between sample analysis.^36^

### 2.6 Instrument conditions

#### 2.6.1 Liquid chromatography

The separation of PPPCPs was carried out using UHPLC (Dionex Ultimate 3000, Thermo Scientific, MA, USA) hyphenated with Q‐Exactive Orbitrap MS (Thermo Scientific, MA, USA). The UHPLC consists of a quaternary solvent manager, degasser, thermostat auto‐sampler, and column oven. The chromatographic separation of the PPPCPs was achieved using Acquity BEH C_18_ column (100 × 2.1 mm, 1.7 μm; Waters, MA, USA). The column and auto‐sampler temperatures were maintained at 35°C and 10°C, respectively, with an injection volume of 10 μL. The mobile phase (A) consisted of 0.05% formic acid in water and mobile phase (B) consisted of 0.05% formic acid in acetonitrile: methanol (50:50, v/v) with a flow rate of 0.3 mL/min was used for the analysis. A linear gradient program started from 2% mobile phase (B) with an initial hold of 0.5 min to a direct increase to 98% mobile phase (B) from 0.5 to 23 min, and hold 98% mobile phase (B) till 26 min, and then decreased to 2% mobile phase (B) in 0.5 min with the column equilibration of 3.5 min with 2% mobile phase (B) with a total run time of 30 min for PPPCPs analysis.

#### 2.6.2 High‐resolution mass spectrometry

The mass identification and quantification of PPPCPs were performed using UHPLC‐Q‐Orbitrap‐MS consisting of a heated electrospray ionization source (HESI), a quadrupole mass filter, higher‐energy collisional dissociation (HCD) cell for highest performance, MS/MS fragmentation, and high‐resolution Orbitrap mass analyzer with resolving power up to 140 000 at *m/z* 200. The HRMS parameters were: capillary temperature of 320°C, heater temperature of 350°C, electrospray voltage of 3.8 kV, S‐Lens RF level at 52 (arb), auxiliary gas (N_2_) at 9 (arb), sheath gas (N_2_) 37 (arb), and micro scans performed at 1 scan/s were used for the analysis. Nitrogen gas with high purity of 99.999% was used for the sheath and auxiliary gases in the ionization source, and also as collision gas in the HCD fragmentation cell. XCalibur 9890 Qual&Quan was used as data acquisition and quantification software.

The HRMS full scan (MS1) was operated in both positive and negative modes in the scan range of 75‐1125 Da at a resolution of 70 000 with maximum injection time of 200 ms, and automatic gain control (AGC) set at 1.0e^5^. All acquisition methods in this study include a full‐scan (MS1) followed by targeted study with parallel reaction monitoring (PRM) MS2 data collection with predefined “inclusion list” that was used in the selection of precursor ions. Table 2 displays the specific normalized collision energy (CE) used for each analyte in PRM mode at a resolution of 17 500 (FWHM at 200 Da) with a maximum injection time set at 100 ms. The AGC target was optimized to 2.0e^4^ with an isolation window of *m/z* 4 for analysis of PPPCPs. The PRM data were acquired in profile mode for full scan analysis and centroid mode for MS/MS analysis.

**TABLE 2:** Q‐Orbitrap‐MS instrument parameters for 26 PPPCPs

Class	Analytes	Mass (*m/z*)	Formula	Mode	RT (min)	Error (ppm)	Collision energy (eV)	MS2 (Ion‐1) (*m/z*)	MS2 (Ion‐2) (*m/z*)
Antiepileptic	Carbamazepine	237.1022	C_15_H_12_N_2_O	+ve	11.3	‐1.02	30	194.0962	192.0805
β‐Blocker	Atenolol	267.1703	C_14_H_22_N_2_O_3_	+ve	4.9	‐2.7	30	190.0859	208.0963
β‐Blocker	Pindolol	249.1568	C_14_H_20_N_2_O_2_	+ve	6.68	‐2.7	30	116.107	172.0752
β‐Blocker	Metoprolol	268.1907	C_15_H_25_NO_3_	+ve	8	‐3	30	159.0798	191.106
β‐Blocker	Propranolol	260.1645	C_16_H_21_NO_2_	+ve	10.1	‐0.91	30	116.1073	183.0802
Opioid	Tramadol	264.1958	C_16_H_25_NO_2_	+ve	8.1	‐3	18	246.1846	231.0436
NSAIDs	Ketoprofen	255.1016	C_16_H_14_O_3_	+ve	13.4	2.3	15	209.0956	105.0336
NSAIDs	Diclofenac	296.024	C_14_H_11_Cl_2_NO_2_	+ve	15.8	‐1.05	20	250.0183	215.0494
NSAIDs	Naproxen	231.1016	C_14_H_14_O_3_	+ve	13.5	‐2.5	10	185.0955	149.023
Steroid	β‐Estradiol	273.1849	C_18_H_24_O_2_	+ve	13	‐1.8	10	107.0491	213.1271
Steroid	Estrone	271.1693	C_18_H_22_O_2_	+ve	13.99	‐1.33	10	253.1584	157.0648
Steroid	Prednisolone	361.201	C_21_H_28_O_5_	+ve	10.4	‐3.1	10	343.1896	325.179
Phthalates	Bis (methyl glycol) phthalate	283.1176	C_14_H_18_O_6_	+ve	11.46	‐3	10	207.0649	147.0801
	Dicyclohexyl Phthalate	331.1904	C_20_H_26_O_4_	+ve	21.59	‐3.2	10	167.0335	149.023
	Dimethyl Phthalate	195.0652	C_10_H_10_O_4_	+ve	11.2	‐2.2	10	163.0385	95.0858
	Dioctyl Phthalate	391.2843	C_24_H_38_O_4_	+ve	25.8	‐3	10	149.023	167.0335
	Dihexyl Phthalate	335.2217	C_20_H_30_O_4_	+ve	23.3	‐3.3	10	149.0231	205.0853
	Diethyl Phthalate	223.0965	C_12_H_14_O_4_	+ve	14.1	‐2.9	10	149.023	177.0542
	Dibutyl Phthalate	279.1591	C_16_H_22_O_4_	+ve	19.3	‐2.5	10	149.0231	205.0854
Parabens	Methylparaben	153.0546	C_8_H_8_O_3_	+ve	9.2	‐1.8	10	121.0284	113.9637
	Ethylparaben	167.0703	C_9_H_10_O_3_	+ve	10.9	‐2.4	10	139.0387	95.0494
	Propylparaben	181.0859	C_10_H_12_O_3_	+ve	12.6	‐2.7	10	139.0387	95.0494
	Butylparaben	193.087	C_11_H_14_O_3_	‐ve	14.2	0.8	10	137.023	93.033
Personal Care Products	Diethanolamine	106.0863	C_4_H_11_NO_2_	+ve	0.78	1.6	30	88.076	70.0657
	Triethanolamine	150.1125	C_6_H_15_NO_3_	+ve	0.8	‐1.9	30	132.1017	132.1017
	Triclosan	286.9439	C_12_H_7_Cl_3_O_2_	‐ve	18.3	2.6	10	154.9899	167.0124

### 2.7 Analytical method validation

The developed method has been validated with respect to linearity, limit of detection (LOD), limit of quantification (LOQ), recovery, and precision as per ICH and SANTE guidelines. The linearity plot was constructed with eight different concentrations (1, 2, 4, 8, 16, 32, 64, and 125 ng/L) for PPPCPs in river water samples (matrix‐matched calibration). The sensitivity of the method was calculated by LOD and LOQ. Recovery study was performed at three different concentrations (2, 30, and 125 ng/L) in the river water samples to evaluate the accuracy of the developed method. Intra‐ and interday precisions were checked by carrying out six independent tests of the sample in a day, for six consecutive days. Matrix effect (ME) was calculated by standard addition method (matrix matched calibration). Further, the parameters like specificity and matrix effect were also assessed. All experiments were performed in triplicate.

## 3 RESULTS AND DISCUSSION

### 3.1 HRMS optimization

The analysis was performed in full‐scan (MS1) and targeted monitoring mode (MS2) for better sensitivity of fragmented ions. The signal to noise (S/N) ratio was always kept at higher than 10 in full scan. The analyte confirmation was carried out using criteria of LC retention time (RT) tolerance of ±2.5% and mass error of ≤5 ppm for the monoisotopic mass in HRMS.^28^, ^37^ The specific fragmented ion for each target analyte was determined by PRM mode (Table 2).

### 3.2 UHPLC optimization

Different mobile phase modifiers were screened, out of which formic acid gave better peak separation, resolution, and sensitivity for the analysis of PPPCPs in river water samples. Milli‐Q water with formic acid (0.05%) was used as mobile phase A, and a mixture of acetonitrile and methanol (1:1, v/v) with formic acid (0.05%) was used as mobile phase B for optimum ionization and separation of the PPPCPs. The C_18_ column showed high‐quality chromatographic separation with symmetrical peaks and less peak tailing for neutral and basic analytes.

### 3.3 SPE sample cleanup

The major part of the study was carried out using SPE HLB cartridge as sorbent for the analysis of PPPCPs.^38^ The pH of the water sample was maintained at 7.0 for obtaining maximum recoveries of PPPCPs. In the SPE method, the elution solvent is also a significant parameter, which extracts all the targeted analytes from the matrix. For maximum efficiency, the elution solvent should have the following characteristics: (a) higher dissolving capability to extract the targeted analytes, (b) higher volatility, and (c) suitable for chromatographic analysis. Based on this criterion, a mixture of dichloromethane (DCM) and methanol (MeOH) in (1:1, v/v) in 10 mL was used as an elution solvent for maximum extraction efficiency of PPPCPs.

### 3.4 Method validation

The developed method was validated as per ICH and SANTE guidelines.^39^, ^40^

#### 3.4.1 Linearity

Analytical method linearity is the ability to produce results that are directly proportional to the analyte concentration in the samples. The method linearity was constructed by 8‐point calibration curve of PPPCPs in river water in the concentration range of 1‐125 ng/L using linear least square method. The coefficient of determination (*R^2^*) was found to be in the range of 0.995‐0.999 for all the selected PPPCPs (Table 3). The linear regression data for the linearity plot show an excellent linear relationship throughout the linearity range.

**TABLE 3:** Method validation parameters for 26 PPPCPs

						% Relative Recovery ± %RSD (n = 6)					
						Intraday			Interday		
Analytes	Linearity*	*R* ^2^	LOD*	LOQ*	ME % ± RSD%	2*	30*	125*	2*	30*	125*
Carbamazepine	2–125	0.996	0.37	1.23	94.46 ± 2.9	91.93 ± 6.4	103. 70 ± 5.2	97.4 ± 4.7	93.93 ± 10.5	107.38 ± 13.0	96.20 ± 8.6
Atenolol	2–125	0.999	0.49	1.61	100.16 ± 8.9	100.48 ± 8.9	102.90 ± 5.7	99.16 ± 8.3	98.64 ± 11.1	97.69 ± 7.6	90.58 ± 11.6
Pindolol	2–125	0.995	0.44	1.46	99.81 ± 8.3	82.49 ± 9.6	96.33 ± 4.6	97.02 ± 8.3	81.77 ± 12.7	93.24 ± 6.0	98.07 ± 13.8
Metoprolol	2–125	0.999	0.47	1.55	90.17 ± 8.3	88.11 ± 8.8	106. 13 ± 6.2	93.15 ± 6.1	96.69 ± 10.1	102.16 ± 10.1	95.52 ± 13.1
Propranolol	2–125	0.999	0.45	1.49	108.92 ± 3.7	91.47 ± 8.6	100.79 ± 4.3	108.70 ± 2.6	84.51 ± 12.3	93.08 ± 8.3	102.08 ± 7.7
Tramadol	2–125	0.998	0.52	1.71	91.38 ± 11.5	94.01 ± 9.6	99.20 ± 5.3	93.09 ± 7.5	82.80 ± 12.5	96.52 ± 6.8	104.64 ± 11.9
Ketoprofen	2–125	0.998	0.39	1.3	103.56 ± 2.5	107.70 ± 6.5	97.19 ± 2.8	99.14 ± 4.9	102.02 ± 7.5	98.74 ± 4.5	99.81 ± 6.1
Diclofenac	1–125	0.996	0.3	0.99	104.98 ± 5.8	107.99 ± 4.8	87.50 ± 7.4	101.50 ± 4.5	102.79 ± 5.5	89.98 ± 12.0	96.13 ± 9.1
Naproxen	1–125	0.999	0.12	0.41	100.85 ± 3.6	92.02 ± 2.2	99.71 ± 1.9	101.82 ± 3.1	99.00 ± 5.9	102.64 ± 3.8	101.38 ± 4.5
β‐Estradiol	1–125	0.999	0.15	0.51	100.70 ± 3.6	75.11 ± 3.6	104.87 ± 1.2	97.46 ± 4.6	79.78 ± 6.4	101.59 ± 2.0	98.92 ± 4.8
Estrone	2–125	0.997	0.36	1.2	99.61 ± 1.1	97.99 ± 6.2	100.68 ± 4.4	104.47 ± 7.3	98.67 ± 6.9	98.63 ± 8.2	104.43 ± 8.0
Prednisolone	2–125	0.997	0.43	1.45	104.71 ± 4.1	87.89 ± 9.3	98.87 ± 7.8	100.30 ± 5.7	89.27 ± 13.3	102.36 ± 11.2	99.75 ± 7.8
Bis (methylglycol) phthalate	2–125	0.998	0.52	1.72	94.31 ± 3.8	101.79 ± 8.4	105.35 ± 3.3	96.98 ± 3.4	97.08 ± 8.8	103.44 ± 5.9	96.88 ± 7.2
Dicyclohexyl phthalate	1–125	0.999	0.3	0.99	83.53 ± 2.1	106.73 ± 4.0	90.98 ± 5.7	86.18 ± 1.9	103.72 ± 5.1	93.52 ± 8.7	84.52 ± 3.8
Dimethyl phthalate	2–125	0.996	0.47	1.56	90.65 ± 2.8	85.78 ± 8.6	101.77 ± 1.7	91.62 ± 4.0	85.61 ± 3.9	103.30 ± 2.2	90.33 ± 8.1
Dioctyl phthalate	2–125	0.999	0.35	1.16	96.35 ± 2.9	89.30 ± 6.5	100.71 ± 3.8	98.38 ± 3.4	101.66 ± 8.1	99.40 ± 6.2	101.53 ± 8.2
Dihexyl phthalate	1–125	0.995	0.31	1.04	101.40 ± 1.2	97.26 ± 5.5	96.60 ± 4.8	102.24 ± 2.7	93.93 ± 6.9	97.23 ± 8.1	104.74 ± 6.1
Diethyl phthalate	2–125	0.999	0.48	1.59	109.79 ± 5.2	114.75 ± 7.5	99.71 ± 2.0	104.97 ± 7.2	91.68 ± 2.7	97.42 ± 3.8	101.99 ± 7.3
Dibutyl phthalate	2–125	0.997	0.41	1.34	95.21 ± 6.2	104.22 ± 9.4	106.00 ± 4.2	94.76 ± 4.5	90.58 ± 7.4	102.94 ± 6.2	97.17 ± 9.1
Methylparaben	1–125	0.999	0.2	0.67	94.31 ± 3.8	98.05 ± 3.8	101.65 ± 2.4	100.89 ± 8.0	98.71 ± 5.8	101.40 ± 3.7	104.86 ± 8.8
Ethylparaben	2–125	0.999	0.38	1.24	100.26 ± 2.4	100.31 ± 6.4	102.99 ± 3.9	96.68 ± 4.7	99.33 ± 6.6	104.60 ± 6.1	101.13 ± 8.9
Propylparaben	1–125	0.996	0.2	0.65	99.13 ± 2.2	101.90 ± 3.2	99.58 ± 2.6	102.15 ± 4.8	100.64 ± 5.5	98.81 ± 4.5	105.83 ± 6.6
Butylparaben	1–125	0.999	0.26	0.85	103.80 ± 5.2	102.89 ± 4.2	103.29 ± 4.4	103.58 ± 5.0	99.73 ± 5.5	100.82 ± 5.4	105.22 ± 5.5
Triethanolamine	1–125	0.999	0.21	0.7	102.87 ± 2.3	99.64 ± 3.7	99.07 ± 4.1	100.9 ± 4.1	101.73 ± 5.2	95.47 ± 6.0	98.89 ± 6.1
Diethanolamine	1–125	0.998	0.26	0.86	106.46 ± 5.8	106.78 ± 4.5	93.82 ± 5.5	106.47 ± 4.6	102.32 ± 6.8	96.57 ± 5.8	105.02 ± 5.5
Triclosan	1–125	0.999	0.25	0.81	100.87 ± 1.9	91.57 ± 4.6	101.50 ± 5.8	101.15 ± 2.3	97.59 ± 7.4	97.21 ± 7.0	100.58 ± 3.8

* Concentration in ng/L; ME, matrix effect; LOD, limit of detection; LOQ, limit of quantification; RSD, relative standard deviation.

#### 3.4.2 LOD and LOQ

LOD is defined as the lowest concentration of the analyte with S/N > 3. It is determined by taking three times the standard deviation of the peak area at the lowest level divided by the slope of the standard addition curve (Equation 1). Ten times the standard deviation of the peak area in the lowest concentration divided by the slope of the standard addition curve gives LOQ of the method (Equation 2). Table 3 lists the LOD and LOQ values of all the PPPCPs estimated by this method and calculated using the following equations.

(1) $$\mathrm{LOD}\;=\;\frac{3\times \;\mathrm{Standard}\;\mathrm{Deviation}}{\mathrm{Slope}}$$

(2) $$\mathrm{LOQ}\;=\;\frac{10\;\times \;\mathrm{Standard}\;\mathrm{Deviation}\;}{\mathrm{Slope}}$$

#### 3.4.3 Method specificity

The method specificity was performed in the real river water samples (with and without spiking of the analytes). The samples without spiking were designated as blank water samples, whereas those spiked with PPPCPs at their LOD levels were identified as spiked water samples (n = 8). In the blank sample, no peaks were observed at the specific retention times of the targeted analytes that shows the absence of analytes in the blank water sample. The spiked water samples displayed peaks indicating that the method is highly specific for the selected PPPCPs.

#### 3.4.4 Recovery

For recovery study, three concentrations (2, 30, and 125 ng/L) were taken: one at LOQ level, second at the middle level, and third at the highest level of the linearity range were spiked in the river water to measure the authenticity of the method. Recoveries were calculated based on Equation 3 and found to be in the range of 75.1‐114.7% (Table 3).

(3) $$Recovery\%=\frac{Spiked\;Conc.-Nonspiked\;Conc.}{Add\;Conc.}\times 100$$

#### 3.4.5 Precision

The precision is the ability of the assay to reliably reproduce the results when sub‐samples were taken from the same specimen. The precision of the measurement was determined by performing six replicates at each concentration (2, 30, and 125 ng/L) in the river water samples for inter‐ and intraday repeatability, and is represented as percent relative standard deviation (%RSD). The precision was found to be in the range of 1.2‐9.6% and 2.0‐13.8% for intra‐ and interday, respectively. The values were found to be within the acceptable criteria as per guidelines (<15% RSD; Table 3).

#### 3.4.6 Matrix effect

Matrix effect is a co‐dependent phenomenon and can affect the ionization efficiency of the analytes, and is evaluated to measure the impact of matrix interferences on the analysis of PPPCPs, and to understand the ion intensity enhancement or suppression. ME can affect the quantification of PPPCPs unless they are diminished or compensated. Matrix‐matched calibration by standard addition method was used to evaluate the ME. The percentage ME is the ratio of matrix slope and solvent slope multiplied by 100 (Equation 4). The matrix slopes obtained by the matrix‐matched calibration and solvent slopes obtained by solvent calibration were used for matrix effect analysis.

(4) $$ME\%=\frac{\mathrm{Matrix}\;\mathrm{slope}}{\mathrm{Solvent}\;\mathrm{slope}}\times 100$$

The ME values >100% indicate ion enhancement, <100% indicate ion suppression, and 100% value shows no matrix interference.^41^ A signal enhancement or suppression effect is considered acceptable if the matrix effect values are in the range of 80‐120%. It means a matrix effect >120% or <80% indicates a strong matrix effec.t^42^

Results were found to be in the range of 83.5‐109.79% for the present method (Table 3). Among pharmaceuticals, propranolol, ketoprofen, diclofenac, naproxen, β‐estradiol, and prednisolone showed ion enhancement, and carbamazepine, metoprolol, tramadol, and estrone indicated ion suppression, whereas atenolol and pindolol did not exhibit substantial matrix interference. Among phthalates, only dihexyl phthalate and diethyl phthalate exhibited ion enhancement, while the other phthalates displayed ion suppression. Methylparaben and propylparaben demonstrated ion suppression, while butylparaben showed ion enhancement effect, whereas ethylparaben showed no matrix interference.

Thus, to compensate for ME suppression and ME enhancement in the analysis of PPPCPs, matrix match calibration was used for analyte quantification and recovery studies. Matrix match calibration eliminates all interferences related to sample and other analytes. Matrix effect helps to provide reliable, accurate, and precise results in the analysis of real samples.

### 3.5 Application of method to real samples

The present method was validated by checking its performance in real samples. The method was applied for the analysis of PPPCPs in real water samples collected from nine sampling points of the River Ganga (Table 4). The method was able to identify and quantify 21 analytes in the concentration range of 0.76‐9.49 ng/L for pharmaceuticals, 1.49‐8.67 ng/L for phthalates, and 0.9‐7.58 ng/L for personal care products. Figure 2A represents the total ion chromatogram (TIC) of all the analytes in a standard mixture at 50 ng/L and Figure 2B shows the identified analytes in the river water samples.

**TABLE 4:** PPPCPs concentrations ± SD in river water samples by UHPLC‐Q‐Orbitrap‐MS

Analytes	Sample 1	Sample 2	Sample 3	Sample 4	Sample 5	Sample 6	Sample 7	Sample 8	Sample‐9
Carbamazepine	3.53 ± 0.8	9.49 ± 6.1	BQL	3.86 ± 2.2	2.48 ± 0.3	2.41 ± 0.5	2.48 ± 0.4	3.72 ± 2.3	1.76 ± 1.0
Atenolol	1.98 ± 1.8	BQL	5.84 ± 3.5	2.33 ± 2.1	BQL	BQL	BQL	BQL	BQL
Pindolol	BQL	BQL	BQL	BQL	BQL	BQL	BQL	BQL	ND
Metoprolol	2.74 ± 0.7	5.94 ± 3.6	9.16 ± 2.9	2.63 ± 1.7	3.78 ± 0.4	4.28 ± 1.5	4.19 ± 3.5	BQL	BQL
Propranolol	1.61 ± 0.3	3.08 ± 2.2	2.90 ± 0.6	BQL	BQL	1.87 ± 1.3	BQL	2.78 ± 2.0	2.28 ± 0.7
Tramadol	BQL	BQL	BQL	BQL	BQL	BQL	BQL	BQL	BQL
Ketoprofen	BQL	ND	ND	BQL	BQL	BQL	BQL	BQL	BQL
Diclofenac	ND	5.12 ± 1.1	1.92 ± 1.2	1.95 ± 1.2	ND	BQL	ND	1.34 ± 1.0	4.45 ± 0.7
Naproxen	BQL	BQL	BQL	ND	ND	ND	ND	ND	ND
β‐Estradiol	0.81 ± 0.1	BQL	0.76 ± 0.3	BQL	BQL	BQL	BQL	ND	ND
Estrone	2.54 ± 0.3	BQL	3.42 ± 1.9	BQL	BQL	BQL	BQL	ND	ND
Prednisolone	BQL	BQL	2.72 ± 0.5	1.90 ± 0.9	BQL	1.90 ± 0.2	2.46 ± 0.2	BQL	1.98 ± 1.3
Bis(methylglycol) phthalate	3.75 ± 1.2	3.21 ± 2.3	6.51 ± 2.3	2.03 ± 1.3	2.18 ± 0.4	1.89 ± 0.2	2.95 ± 1.2	3.82 ± 2.5	2.75 ± 1.7
Dicyclohexyl phthalate	3.79 ± 0.9	ND	ND	4.06 ± 3.5	4.59 ± 1.4	2.72 ± 1.8	5.42 ± 0.6	2.84 ± 1.5	1.49 ± 0.8
Dimethyl phthalate	ND	ND	ND	ND	ND	ND	ND	5.12 ± 2.5	ND
Dioctyl phthalate	ND	ND	ND	BQL	ND	ND	BQL	ND	ND
Dihexyl phthalate	BQL	1.73 ± 2.0	8.67 ± 0.4	BQL	BQL	BQL	BQL	BQL	BQL
Diethyl phthalate	BQL	BQL	2.88 ± 0.1	BQL	BQL	BQL	BQL	BQL	BQL
Dibutyl phthalate	3.31 ± 0.4	7.72 ± 5.3	4.96 ± 1.5	6.22 ± 4.1	3.57 ± 0.2	5.11 ± 0.8	2.89 ± 0.6	2.81 ± 1.3	1.78 ± 0.8
Methylparaben	BQL	BQL	BQL	1.82 ± 0.8	2.01 ± 0.4	3.00 ± 2.0	1.75 ± 0.1	0.95 ± 1.1	0.97 ± 1.1
Ethylparaben	1.59 ± 1.3	BQL	BQL	ND	ND	ND	ND	BQL	BQL
Propylparaben	2.11 ± 0.5	1.50 ± 0.1	2.69 ± 0.9	1.69 ± 1.8	2.14 ± 0.3	2.08 ± 0.4	2.14 ± 0.4	1.71 ± 1.1	1.93 ± 1.0
Butylparaben	2.45 ± 0.5	ND	1.56 ± 0.6	0.92 ± 0.4	1.37 ± 0.5	3.04 ± 1.0	1.61 ± 0.1	ND	ND
Triethanolamine	2.17 ± 2.0	ND	ND	BQL	BQL	ND	ND	ND	ND
Diethanolamine	BQL	ND	ND	0.90 ± 0.6	BQL	1.73 ± 1.3	BQL	ND	ND
Triclosan	2.59 ± 0.9	7.58 ± 4.7	5.47 ± 2.4	BQL	BQL	BQL	BQL	3.45 ± 3.8	3.52 ± 4.0

ND, not detected; BQL, below quantitation limit.

FIGURE 2
**A**, Total ion chromatogram of standard PPPCPs (50 ng/L) obtained from UHPLC–Q‐Orbitrap‐MS analysis. **B**, Total ion chromatogram of analysis of PPPCPs in the river water samples

**Figure:**
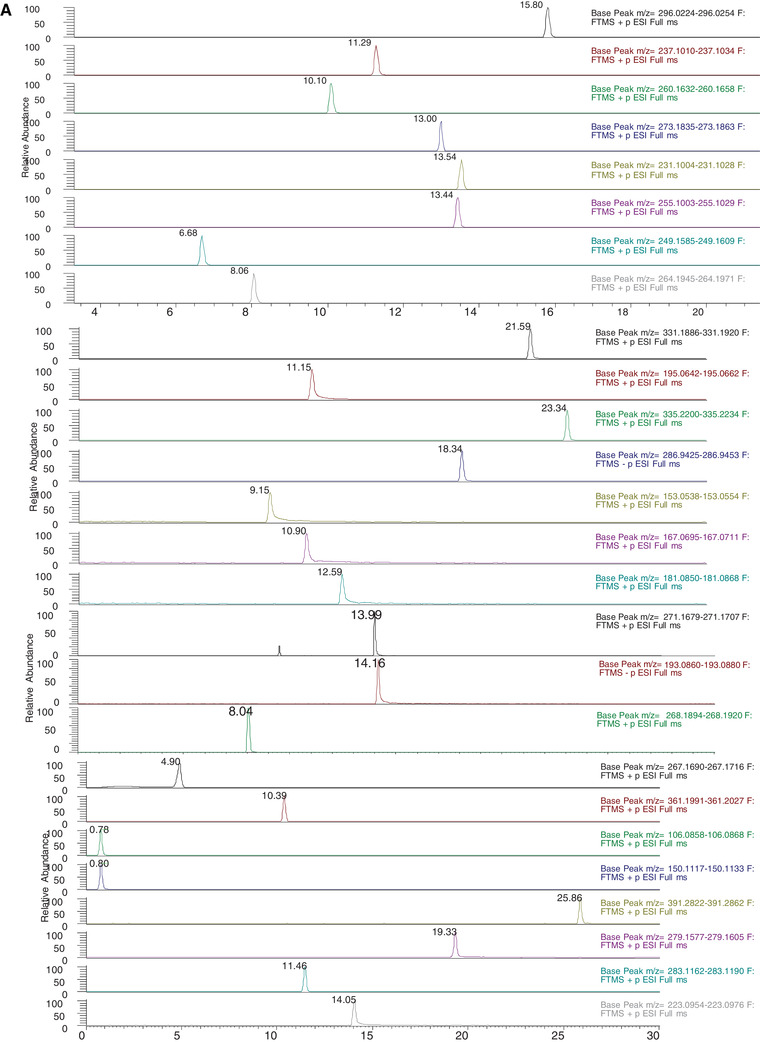

**Figure:**
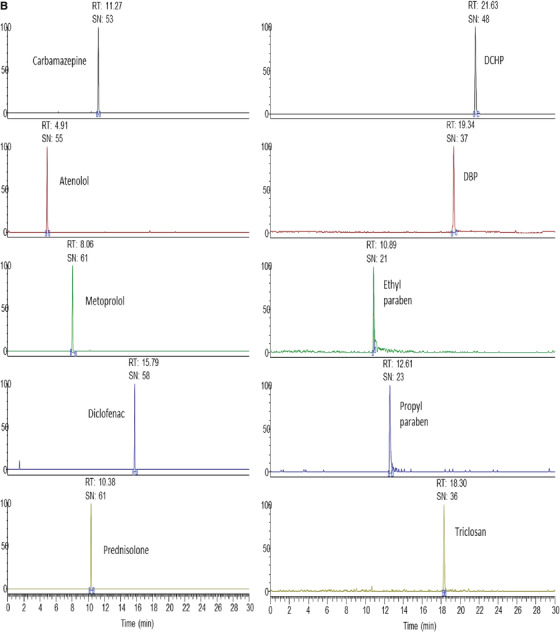

Among pharmaceuticals, β‐blockers like atenolol, metoprolol, and propranolol were found in water at concentrations of 5.84, 9.16, and 1.61‐3.08 ng/L, respectively, while pindolol was detected but not quantified at LOQ level. Carbamazepine (an antiepileptic medication used to treat epilepsy and bipolar disorders) that is one of the highest consumed drugs in India was observed in the concentration range of 1.76‐9.49 ng/L in some of the samples.^43^

Diclofenac, a non‐steroidal anti‐inflammatory drug (NSAID), was found in the concentration range of 1.34‐5.12 ng/L. The values reported for diclofenac are low in the analyzed samples in comparison to those obtained from previous reports.^44^ At most of the sampling points, ketoprofen and naproxen were either not detected or were below quantitation limits. The compounds like β‐estradiol, estrone, and prednisolone were found in the range of 0.76‐3.42 ng/L.

The phthalates are considered as potential endocrine‐disrupting chemicals (EDCs) in humans and cause numerous health disorders.^45^ Dihexyl phthalate (DHP) and dibutyl phthalate (DBP) used as regular plasticizers were found at the concentrations of 8.67 and 7.72 ng/L, respectively, followed by bis(2‐methoxyethyl) phthalate (DMEP) (6.51 ng/L). Dicyclohexyl phthalate (DCHP) and diethyl phthalate (DEP) were found in the range of 1.49‐5.42 ng/L, whereas dimethyl phthalate (DMP) was found in one sample at a concentration of 5.12 ng/L. Dioctyl phthalate (DOP) was found at below quantification level (BQL) at two sampling points, due to its low solubility in water. The parabens (methyl, ethyl, propyl, and butyl) are a class of preservatives found in most of the cosmetics and food commodities and were found at low concentrations in the range of 0.92‐3.04 ng/L.^46^ The other personal care products, triclosan, triethanolamine, and diethanolamine were found in the range of 0.90‐7.58 ng/L.^47^

Out of 26, 21 analytes were detected in the river water samples in low concentrations. However, this study emphasizes the need for continuous cleanup/remediation measures to effectively remove the PPPCPs from river water samples.

### 3.6 Comparison of present method with earlier reported methods

The present method was found to be superior to earlier reported methods for the analysis of PPPCPs including phthalates with respect to linearity, LOD, LOQ, and recovery (Table 5). The method linearity of the present study was in the range of 1–125 ng/L and the values of LOD and LOQ were also low in the present study, which shows that the present study is better than those reported earlier.

**TABLE 5:** Comparison of the SPE method with earlier reported methods

Serial number	Similar analytes	Matrix	Extraction Method	Instrument	Linearity	LOD	LOQ	Recovery	References
1	Estrone Carbamazepine Diclofenac Gemfibrozil Ketoprofen Naproxen Triclosan	1.0 g of Sewage sludge	Ultrasonic extraction	GC‐MS/MS	2‐2000 ng/g	1.6‐11 ng/g	5.4‐39 ng/g	75.3‐95.5%	^16^
2	Diclofenac Estrone 17β‐Estradiol	5.0 g of Sediment	SPE	UPLC‐MS/MS	1‐200 ng/ mL	0.02–0.81 ng/g	–	78‐108%	^21^
3	Metoprolol Propranolol Tramadol Carbamazepine Naproxen β‐Estradiol	250 mL of Effluent and surface water	SPE	UHPLC‐Q‐Orbitrap‐MS	1‐1500 ng/mL	0.02–1.21 ng/mL	0.07–4.05 ng/mL	76‐104%	^30^
4	Carbamazepine Atenolol Pindolol Metoprolol Propranolol Tramadol Ketoprofen Diclofenac Naproxen β‐Estradiol Estrone Prednisolone Methylparaben Ethylparaben Propylparaben Butylparaben Triethanolamine Diethanolamine Triclosan including 7 phthalates	3 litre of River Water	SPE	UHPLC‐Q‐Orbitrap‐MS	1‐125 ng/L	0.12‐0.52 ng/L	0.41‐1.71 ng/L	75‐115%	Present Study

## 4 CONCLUDING REMARKS

A sensitive and efficient analytical method has been developed for the analysis of 26 PPPCPs including phthalates in Ganga River water using SPE‐UHPLC‐Q‐Orbitrap‐MS. The method validation results were: linearity (1‐125 ng/L), LOD (0.12–0.52 ng/L), LOQ (0.41–1.71 ng/L), recovery (75.1‐114.7%), precision (1.2‐9.6% in intraday and 2.0‐13.8% in interday), and matrix effect (83.5‐109.79). The PPPCPs were found in the concentration range of 0.76‐9.49 ng/L, 0.9‐7.58 ng/L, and 1.49‐8.67 ng/L for pharmaceuticals, personal care products, and phthalates, respectively, in Ganga River water samples. The developed and validated method was able to identify and quantify multiclass PPPCPs in water samples using SPE‐UHPLC‐Q‐Orbitrap‐MS with acceptable precision and accuracy, and would be useful for routine environmental monitoring studies.

## CONFLICT OF INTEREST

The authors declare no conflict of interest.

## Data Availability

The data that support the findings of this study are available in the tables and figures of this article.
